# The N-Glycosylation Modification of LHBs (Large Surface Proteins of HBV) Effects on Endoplasmic Reticulum Stress, Cell Proliferation and its Secretion

**DOI:** 10.5812/hepatmon.12280

**Published:** 2013-09-07

**Authors:** Wenxiang Liu, Yongmei Cao, Tao Wang, Guoan Xiang, Jiangyang Lu, Jinqian Zhang, Peng Hou

**Affiliations:** 1 Department of Gastroenterology and Hepatology, the First Affiliated Hospital of Chinese PLA General Hospital, Beijing, China; 2 International Mongolian Hospital, Hohhot of Inner Mongolia, Hohhot, China; 3 Department of General Surgery, the Second People's Hospital of Guangdong Province, Guangzhou, China; 4 Department of Pathology, the First Affiliated Hospital of Chinese PLA General Hospital, Beijing, China; 5 Institute of Infectious Diseases, Beijing Ditan Hospital, Capital Medical University, Beijing, China

**Keywords:** Glycosylation, Hepatitis B Virus, Endoplasmic Reticulum Stress, Cell Proliferation

## Abstract

**Background:**

The mutations of LHBs in pre-S, especially in pre-S2, are definitive in hepatocellular carcinoma (HCC) associated with HBV. However, the mechanisms of the N-glycosylation modification in LHBs are unclear. The N-glycosylation modification of LHBs affects Endoplasmic Reticulum stress, cell proliferation and its secretion which was further studied.

**Objectives:**

The objectives of our studies was to indentified that modification of LHBs by N glycosylation modulate their secretion, affect ER stress or expression of cycling, cell cycle and proliferation.

**Materials and Methods:**

The LHBs was mutated; then expression of proteins related to endoplasmic reticulum stress and EAED path of L02 cells affected by LHBs and its mutations was evaluated. LHBs proteins bound to multiubiquitin chains and its glycosylation motif were studied. The subcellular localization and secretion of LHBs and its mutations were identified. The effect on cell cycle and proliferation by LHBs and its mutations were detected.

**Results:**

These data demonstrated that the N-glycosylation motifs of LHBs were associated with ER stress. The N15S, N123S, and N177S mutated LHBs proteins could induce overexpression of EDEM in L02 cells. LHBs and its mutated proteins contained p62-derived UBA domain, which could affect expression of cyclins. The subcellular localization of LHBs in endoplasmic reticulum was similar to its mutations. The secretion of LHBs was blocked by N320K mutation, which could induce an increase in G1 phase and inhibition of S phase, and inhibited mitotic entry.

**Conclusions:**

In conclusion, our studies powerfully demonstrated that modification of LHBs by N glycosylation could modulate their secretion, affect ER stress or expression of cycling, cell cycle and proliferation. The N320K may be the key sites N-linked glycosylation modification of LHBs. It may be a mechanism of HBV-induced HCC.

## 1. Background

HBV (Hepatitis B virus) is the most common infectious agent affecting the liver world-wide (1, 2). Patients with chronic hepatitis B are susceptible to develop chronic hepatitis, cirrhosis, especially HCC (3-5). The hepatitis B virus (HBV) is coated with LHBs (large surface proteins of HBV), MHBs (middle surface proteins of HBV), and SHBs (small surface proteins of HBV) (6-8).

HCC was significantly related to two pre-S deletion mutants of LHBs (9-12), which could pile up in endoplasmic reticulum (ER). They cause predominant stress response in ER, and then induce oxidative stress and DNA damage (13-15). The expression of pre-S2 mutant LHBs in hepatocytes cluster into groups and exhibit clonal expansion and growth advantage. According to these studies, mutant of LHBs is correlated with hepatocellular carcinogenesis, especially in pre-S2 mutant of HBV.

N-glycosylation is the key modification type of proteins, and modulates their crucial functions in cells (16, 17). Proteins are added with N-linked glycans, presynthesized oligosaccharides in lumen of ER. The oligosaccharyltransferase (OST) complex catalyzed reaction of N-glycosylation, the target sequence is N-X-T/S, glycan unit is linked to the asparagine residue (16, 17). Carsten et al. found that N-glycosylation modification is present on LHBs too (18).

We previously demonstrated that pre-S2 LHBs could interact with C53; it is an unprecedented regulator of checkpoint response (19). Then pre-S2 LHBs could enhance the activation of Cdk1 and proliferation of liver cells, which is related to HCC (19). Then, the N-glycosylation Modification of LHBs affects Endoplasmic Reticulum Stress, cell proliferation and its secretion that was further studied.

## 2. Objectives

The objectives of our studies was to indentified that modification of LHBs by N glycosylation modulate their secretion, affect ER stress or expression of cycling, cell cycle and proliferation.

## 3. Materials and Methods

### 3.1. Materials

L02 was purchased from the American Type Culture Collection (ATCC) (USA). Cell culture medium Dulbecco’s modified Eagle’s medium (DMEM), penicillin, streptomycin medium supplement, glutamine and fetal bovine serum (FBS) were obtained from Gibco life Technologies (UK). Western blotting materials were purchased from Roche Applied Sciences (USA). cDNA Synthesis Kit was purchased from Fermentas, EU. SYBR green DNAPCR Master Mix was purchased from the Applied Biosystem (ABI) Company (Foster City, CA, The USA). Site-directed mutagenesis was used the TransformerTM Site-Directed Mutagenesis kit (Clontech). All antibodies were obtained from the Santa Cruz (The USA). Protein G Plus/Protein AAgarose Suspension was obtained from Calbiochem (the USA). LHBs ELISA Kit was purchased from Auszyme (Abbott, The USA). Lipofectamine 2000 was purchased from Invitrogen (USA). The flow cytometer was purchased from BD (The USA). Dimethyl sulfoxide (DMSO) and all other chemicals were obtained from the Sigma-Aldrich (The USA).SPSS 11.0 software (USA) was used for the statistical analyses.

### 3.2. Plasmid Construction

LHBs were amplified from serum of a patient with chronic hepatitis B (subtype B), using upstream Primer (5'-cggaattccgatgggaggttg-3') and downstream primer (5'-ccaagcttggtcaaatgtataccc-3'). The restriction sites, EcoRI and Hind III, were incorporated into the primer sequences for cloning purposes. The PCR reaction conditions were initial denaturing at 95 °C for 5 min, denaturing at 94 °C for 40 sec, annealing at 58 °C for 45 sec, and extension at 72 °C for 1 min. A total of 40 reaction cycles was performed. The PCR product was purified and cloned into pCDNA-3.1(-) vector, then obtained the plasmid pCDNA-3.1(-) - LHBs.

Informed consent was obtained from each patient included in the study, and the study protocol conformed to the ethical guidelines of the 1975 Declaration of Helsinki as reflected in a priori approval by our institution's human research committee.

LHBs was cloned into pEGFP-C1 expression vector using upstream Primer (5'-aagcttatgggaggttgg-3') and downstream primer (5'-ggatccaatgtatacccaaagac -3'). The restriction sites, BamHI and Hind III, were incorporated into the primer sequences for cloning purposes. The PCR reaction conditions were initial denaturing at 95 °C for 5min, denaturing at 94 °C for 40 sec, annealing at 58 °C for 45 sec, and extension at 72 °C for 1 min. A total of 40 reaction cycles was performed. The PCR product was purified and cloned into pEGFP-C1vector, then obtained the plasmid pEGFP-C1- LHBs.

CALR was cloned using forward primer (5’- ctcgagatggcgggatcc -3’) and reverse primer (5’- ggtaccggaaagaattttttggc -3’). The restriction sites, Xho I and Kpn I, were incorporated into the primer sequences for cloning purposes. The PCR reaction conditions were initial denaturing at 95 °C for 5 min, denaturing at 94 °C for 40 sec, annealing at 54 °C for 40 sec, and extension at 72 °C for 1 min. A total of 40 reaction cycles was performed. The PCR product was purified and cloned into the pDS-RED1-N1 expression vector, then obtained the plasmid pDS-RED1-N1- CALR.

### 3.3. Mutation of LHBs

**Figure 1.**
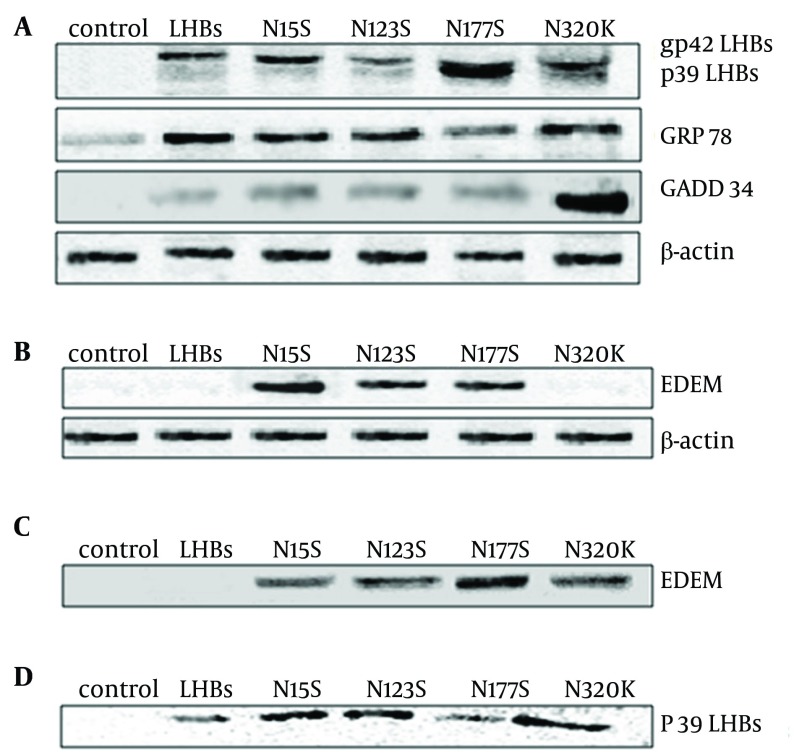
Western Blot Analyses for the Expression of Proteins Associated With ER Stress (A), the Expression of EDEM Associated With EAED Path (B). Co-Immunoprecipitation of LHBs or Mutation With EDEM (C). LHBs Proteins Bind to Multi-Ubiquitin Chains (UW8860) (D) (A) Blots were probed with anti- LHBs, GRp78 and GADD34 antibodies. (B) Blots were probed with anti- EDEM antibodies. The bands of blots were EDEM (74KDa). (C) Blots were probed with anti-EDEM antibodies. The bands of immunoprecipitation were EDEM (74 kDa). (D) Mixed ubiquitin chains (product code. UW8860; 1 ug) were diluted in 50 mM Tris, 0.1% BSA, pH 7.5 (50µL) and loaded on to washed UBA-agarose beads (product code UW9010) (10µL). Following incubation UBA-agarose beads with proteins from cells respectively, bound proteins were occasional agitated at room temperature for 20 minutes, and then were eluted in SDS PAGE loading buffer (50µL). After run on an SDS-PAGE gel before being transferred for western blot, blots were probed with anti- LHBs antibodies. The bands were LHBs p39 (39 kDa)

Method for site-directed mutagenesis of LHBs was based on the manual of the Transformer TM Site-Directed Mutagenesis kit. The N-glycosylation sites were predicted, and the mutated base and amino acid sequence of the LHBs protein are showed in Figure 1. The selection primer was 5'-caagtgtatcataggccaagtacgccc -3' (nucleotides (nts) 31-57). The N15S mutation was introduced with the mutagenic primer 5'-ggcatggggacaagtctttctgttccc-3' (nucleotides (nts) 31-57). The N123S mutation was introduced with the mutagenic primer 5'- gccatgcagtggagctccaccacattc-3’ (nts 355-381). The N177S mutation was introduced with the mutagenic primer 5'-cgaacatggagagcacaacatcagg-3' (nts 518-542). The N320K mutation was introduced with the mutagenic primer 5'-cttcggacggaaagtgcacttgtattc-3’ (nts 947-973). 

The resulting product was cut with SacI. The EcoR I-Hind Ш-fragment containing the N15S, N123S, N177S and N320K mutations were subcloned into the pCDNA-3.1(-) vector and pEGFP-C1.

### 3.4. Endoplasmic Reticulum Stress Evaluation

For determining the role of LHBs or mutation of LHBs in endoplasmic reticulum stress, we cultured L02 cell for 24 hrs, then transfected with pCDNA3.1 (-), pCDNA3.1 (-)-LHBs, pCDNA3.1 (-)-N15S, pCDNA3.1 (-)-N123S, pCDNA3.1 (-)-N177S, and pCDNA3.1 (-)-N320K respectively. Plates were sealed and incubated for 48 hrs at 37℃. Total proteins were extracted from the separated L02 cells respectively. The expression of GADD34 and GRP78 were detected by western blot respectively.

### 3.5. Co-Immunoprecipitation

L02 cells were grown on culture in 6-well plates for 48 hrs; cells were lysed in lysis buffer with Phenylmethanesulfonyl fluoride (PMSF). The extract was centrifuged, then each supernatant was added 50 μL of Protein G Plus/Protein AAgarose Suspension for 4 hrs at 4°C. The supernatant was added respectively, 10 μL antibody rotated mildly overnight at 4°C, then 60 μL Protein G Plus/Protein A Agarose Suspension was added, and rotated mildly for 9 h at 4°C, centrifuged for 5 min at 4°C. Immunoprecipitates were washed three times in lysis buffer, and then further examined by western blot.

### 3.6. LHBs Proteins Bind to Multi-Ubiquitin Chains

L02 cells were harvested after culturing for 24 hrs, then transfected with pCDNA3.1 (-), pCDNA3.1 (-)-LHBs, pCDNA3.1 (-)-N15S, pCDNA3.1 (-)-N123S, pCDNA3.1 (-)-N177S, and pCDNA3.1 (-)-N320K respectively as described in the above methods. After 48 hrs, the total proteins of cells were extracted respectively.

Mixed ubiquitin chains (product code. UW8860; 1 ug) were diluted in 50 mMTris, 0.1% BSA, pH 7.5 (50µL) and loaded on washed UBA-agarose beads (product code UW9010) (10µL). Following incubation UBA-agarose beads with proteins from cells respectively, bound proteins were occasional agitated at room temperature for 20 minutes, and then were eluted in SDS PAGE loading buffer (50µL). After run on an SDS-PAGE gel before being transferred for Western blotting, blots were probed with anti- LHBs antibodies. The bands were LHBs p39 (39 kDa).

### 3.7. Treatment with ConA or LCA Column

L02 cells were harvested after culturing for 24 hrs, then transfected with pCDNA3.1 (-), pCDNA3.1 (-)-LHBs, pCDNA3.1 (-)-N15S, pCDNA3.1 (-)-N123S, pCDNA3.1 (-)-N177S, and pCDNA3.1 (-)-N320K respectively as described in the above methods. After 48 hrs, the total proteins of cells were obtained and bound to Con A or LCA Sepharose CL-4B column respectively. Then the proteins bound to Con A or LCA column were eluted and run on an SDS-PAGE gel before being transferred for Western blot. Blots were probed with anti-LHBs antibodies.

### 3.8. Glycosidase Treatment

Lysates of cells were boiled for 10 min with denaturing buffer (0.5% SDS, 40 mM DTT). Then the above lysates were incubated for 1 h at 37°C with 500 U N-Glycosidase F (PNGase F) (New England Biolabs). Then the proteins were run on an SDS-PAGE gel before being transferred for Western blot. Blots were probed with anti-LHBs antibodies.

### 3.9. The Subcellular Localization of LHBs and its Mutations

The plasmids pEGFP-C1-LHBs (or pEGFP-C1-N15S, pEGFP-C1-N123S, pEGFP-C1-N177S, pEGFP-C1-N320K) and pDS-RED1-N1-CALR were cotransfected into L02 cells. After transfection for 48 hrs, cells were treated with 4% paraformaldehyde (PFA) for 10 min, washed 3 times with PBS, and stained with 0.1μg/mL 4',6-diamidino-2-phenylindole (DAPI) for 30min at 37℃. Those were observed with Zeiss LSM510 confocal laser scanning microscope.

### 3.10. The Detection Secretion of LHBs and its Mutations

The supernatants of cultured cell were collected and detected using LHBs ELISA Kit (Auszyme, Abbott). The method was according to the kit instruction manual.

### 3.11. Cell Cycle Analysis

Cells were dissociated with trypsin, washed, and resuspended in PBS as a single-cell suspension. Cells were fixed in 70% ethanol overnight, stained with propidium iodide (25 ug/ml), and incubated for 30 min at 37°C with RNase A (20 ug/ml). The cells group treated with PBS was used as the controls. Cells were assessed by flow cytometer and the results were analyzed with Modifit software. The DNA content of the cells was then evaluated by fluorescence-activated cell sorting with a FACSCalibur (BD Immunocytometry Systems).

### 3.12. Cell Synchronization, BrdU Labeling and Mitotic Index

To avoid potential carry-over effects of plasmids transfection-induced cell cycle defects in the previous cycle on the following mitotic entry during the next cycle, plasmids were transfected into cells during the interval between the two thymidine blocks, so that we were able to evaluate direct impact of LHBs and its mutations on mitotic entry. Cells were synchronized by double thymidine block. Briefly, cells were plated at 40% confluency and arrested with 2 mM thymidine. After 19 hrs incubation, cells were washed 4 times with fresh medium and transfected with plasmids using Lipofectamine 2000 (Invitrogen). After incubation with DNA-lipid mixture for 3 hrs, cells were washed twice and incubated in fresh medium for additional 5 hrs. Subsequently, cells were cultured in medium containing 2 mM thymidine and 2 ug/ml puromycin for the second arrest and drug selection. After 16 hrs incubation, cells were released into the cell cycle by incubation in fresh medium. Cells were collected or fixed at indicated time points and subjected to specific analyses.

BrdU labeling was used to evaluate DNA synthesis. After released from the second thymidine arrest at indicated time points, cells grown in 12-well plate were pulse labeled with BrdU (50 uM) for 30 min. After three washes of PBS, cells were fixed with 1 ml of Carnoy’s fixative (3 parts methanol 1:1 part glacial acetic acid) at -20°C for 20 min, and followed by three washes of PBS. Subsequently, DNA was denatured by incubation of 2M HCl at 37°C for 60 min, followed by three washes in borate buffer (0.1 M borate buffer, pH 8.5). After incubation with the blocking buffer, cells were stained with anti-BrdU antibody (BD Biosciences, 1: 100) overnight at 4°C. After washes in PBS, cells were incubated with Texas Red-conjugated anti-mouse goat IgG for 30 min at RT. After washes, cells were mounted and BrdU positive cells were manually scored under immunofluorescence microscope.

### 3.13. Western Blot Analysis

Cells were lysated with Lysate buffer. Samples were loaded on a 12% SDS-PAGE gel, and transferred to PVDF membranes. Membranes were incubated overnight at 4 ℃with rabbit polyclonal anti-GRp78 (1:500), GADD34 antibody (1:1,000 dilution), β-actin (1:1,000), Cyclin A (1:1,000), Cyclin B1 (1:1,000), Cyclin D1 (1:1,000), Cyclin E (1:1,000), LHBs (1:1,000), EDEM (1:1,000).

### 3.14. Statistics

Using SPSS 11.0 software, a 2-tailed Student’s t test was used to compare the statistical difference between the two groups, and a 1-way ANOVA Newman-Keuls Multiple Comparisons Test was used to compare the differences between the three groups or more. Probabilities of 0.05 or less were considered to be statistically significant.

## 4. Results

### 4.1. Detection of LHBs Mutants

In previous works we have focused on the interaction between pre-S2 LHBs and CDK5RAP3 that is the important mechanism about HBV-induced HCC (19). To analyze modification of N-glycosylation of mutated LHBs effects on the ER stress, the sequence of LHBs had been mutated. The N15S, N123S, N177S and N320K LHBs mutation were determined by nucleotide and amino acid sequences analysis (Supplement 1).

### 4.2. The N-Glycosylation Motifs of LHBs Effects on ER Stress

LHBs present unique doublet types, non-glycosylated LHBs (p39, 39-kDa) and single-glycosylated (gp42, 42-kDa). It is due to posttranslational N-glycosylation in S domain of LHBs (Figure 1A). 

Because LHBs protein is localized in ER, we further determined whether the mutation protein of LHBs at N15S, N123S, N177S and N320K was associated with ER stress. The expressions of GADD34 and GRp78 were detected by western blot analysis. In contrast to the markers of ER stress, the expression of GADD34 and GRp78 was significantly upregulated in L02 cells transfected with LHBs and its mutations compared to control, especially N320K mutation (Figure 1A). But, the expression of GADD34 and GRp78 was significantly down-regulated in L02 cells transfected with N177S mutation compared to those of transfected with LHBs and other mutations. These data demonstrated that N-glycosylation modification sites of LHBs were related to ER stress, especially in N177S and N320K N-glycosylation (Figure 1A). 

### 4.3. The Expression of EDEM in EAED Path Associated with LHBs Mutation

Two reports in Science ( 20 , 21 ) have helped to solidify the role of the mammalian protein EDEM (ER degradation-enhancing α-mannosidase-like protein), which is the key receptor associated with quality control in responsible to destruction of malfolded proteins. We further studied the association between N-linked glycosylation modification of LHBs mutation protein and EDEM. Our results showed that the N15S, N123S and N177S mutated LHBs proteins could induce overexpression of EDEM in L02 cells (Figure 1B). The N15S, N123S, N177S and N320K LHBs mutation proteins could interact with EDEM protein in L02 cells, especially N177S, but not LHBs (Figure 1C). 

### 4.4. The UBA Sequence Motifs of LHBs Mutation

After run on an SDS-PAGE gel before being transferred for western blot, blots were probed with anti-LHBs antibodies. The bands were LHBs p39 (39 kDa). The study indicated that LHBs and its four mutated proteins were able to bind to ubiquitin-binding protein p62 multiubiquitin chains (Figure 1D). But, no bands were found while proteins were loaded on washed UBA-agarose beads (product code UW9440, UW9445, UW9700). 

It confirmed that LHBs and it four mutated proteins contained p62-derived UBA domain, especially in N15S, N123S and N320K mutations, but not hHR23B-derived UBA2 domain, NBR1-derived UBA domain and NUB1/NUB1L UBA domain.

### 4.5. The N-Glycosylation Motifs of LHBs Effects on Expression of Cyclins

To determine the effect on cyclins related to N-linked glycosylation modification of LHBs, the expressions of cyclins were detected by western blot analysis.

**Figure 2.**
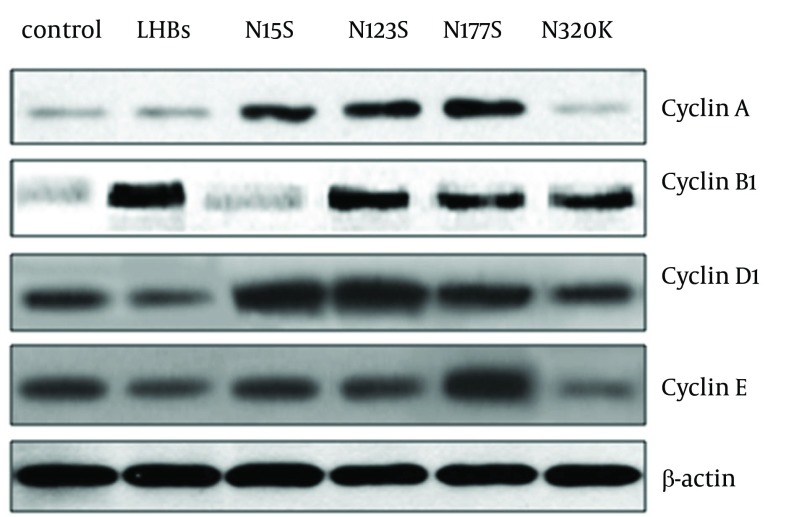
The Effects of LHBs and its Mutations on the Expression of Cyclins (cyclin A, B1, D1 and E) in L02 Cells L02 cells were harvested after culturing for 24 hrs, then transfected with pCDNA3.1 (-), pCDNA3.1(-)-LHBs, pCDNA3.1(-)-N15S, pCDNA3.1(-)-N123S, pCDNA3.1(-)-N177S, and pCDNA3.1(-)-N320K respectively as described in Materials and Methods.After 48 hrs, proteins of cells were extracted and then run on an SDS-PAGE gel before being transferred for western blot. Blots were probed with anti- cyclin A, B1, D1, E antibodies. The bands were cyclin A (54kDa), cyclin B1 (55kDa), cyclin D1 (38kDa) and cyclin E (53 kDa).

Our results showed that the N15S, N123S and N177S mutated LHBs proteins could induce overexpression of cyclinA in L02 cells. The LHBs and N123S, N177S, N320K LHBs mutation proteins could induce overexpression of cyclin B1 in L02 cells, especially in LHBs. The N15S, N123S and N177S LHBs mutation proteins could induce overexpression of cyclin D1 in L02 cells, especially in N15S and N123S LHBs mutations. The N320K LHBs mutation proteins could induce overexpression of cyclin E (Figure 2). 

### 4.6. LHBs and its Mutations Affected Cell Cycle

The effect of overexpression of LHBs and its mutations on cell cycle distribution was determined in L02 cells by flow cytometry. LHBs and its mutations induced an increase in G1 phase and inhibition of S phase, especially in N320K (Supplement 2A).

### 4.7. LHBs and its Mutations Inhibit Mitotic Entry

**Figure 3.**
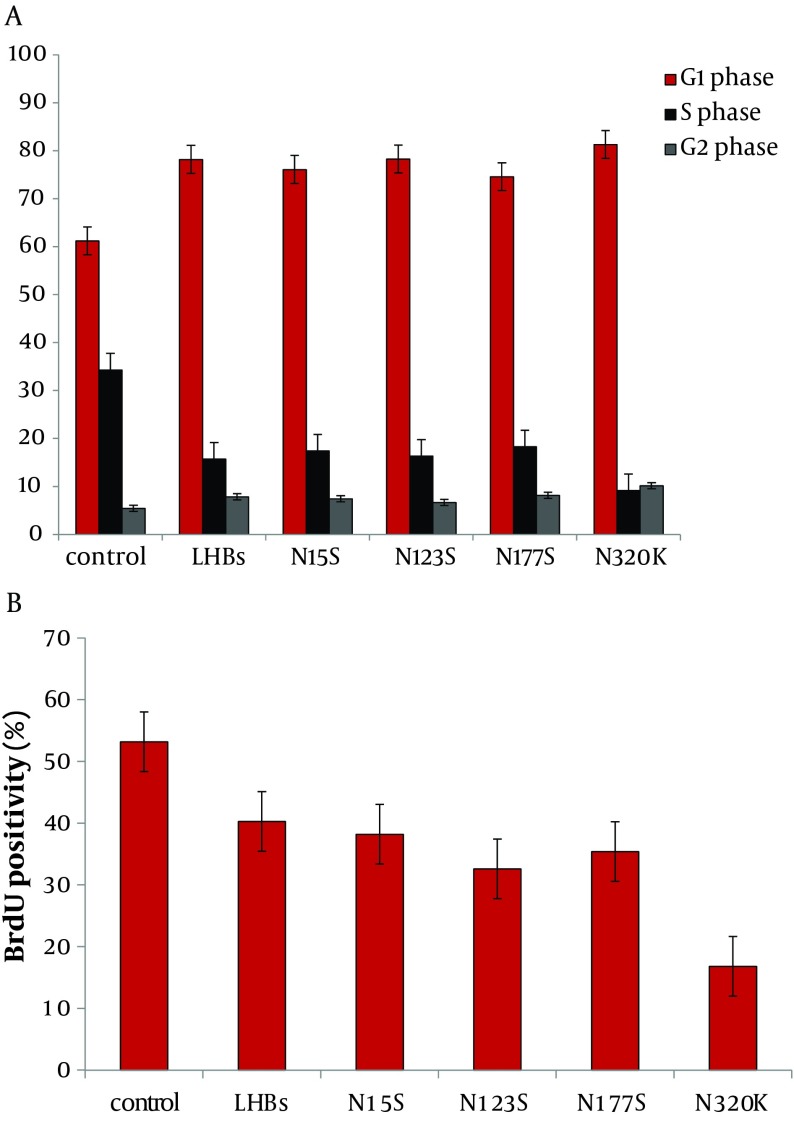
LHBs and Its Mutations Affect Cell Cycle and Inhibit Mitotic Entry L02 cells were transfected with pCDNA3.1 (-), pCDNA3.1 (-)-LHBs, pCDNA3.1 (-)-N15S, pCDNA3.1 (-)-N123S, pCDNA3.1 (-)-N177S, and pCDNA3.1(-)-N320K respectively as described in Materials and Methods. The results showed that LHBs and its mutations induced an increase in G1 phase and inhibition of S phase, especially N320K. After 48 hrs, incorporation of BrdU into the control, accumulation of mitotic L02 cells was significantly delayed in cells treated with LHBs and its mutations, especially N320K. **P < 0.01

Cells were synchronized at the G1/S boundary by double thymidine block, and then released into mitosis. After 24 hrs, BrdU was added into the medium at indicated time points to evaluate DNA synthesis. As shown in Supplement 2, incorporation of BrdU into the control, accumulation of mitotic L02 cells was delayed by LHBs and its mutations, especially in N320K (Figure 3B). 

### 4.8. The N-Glycosylation Motifs of LHBs

To test for Nglycosylation, lysates of LHBs-expressing L02 cells were treated with Con A, LCA and Peptide: N-Glycosidase F (PNGase F) respectively. 

**Figure 4.**
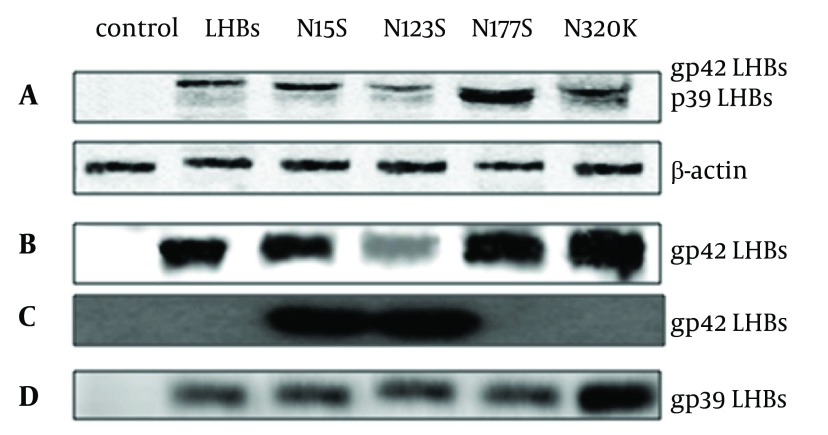
Western Blots Analysis for the Expression of LHBs (A), LHBs Bind to Con A (B), LHBs Bind to LCA (C) and Treated With PNGase F (D) L02 cells were harvested after culturing for 48 hrs, then transfected with pCDNA3.1 (-), pCDNA3.1 (-)-LHBs, pCDNA3.1(-)-N15S, pCDNA3.1(-)-N123S, pCDNA3.1(-)-N177S, and pCDNA3.1(-)-N320K respectively as described in Materials and Methods. After 48 hrs, proteins were obtained then the proteins were run on an SDS-PAGE gel and transferred for western blotting. Blots were probed with anti-LHBs antibodies. The bands of blots were LHBs gp42 (42KDa).

As shown in Figure 4B, LHBs and its four mutations were N-glycoproteins, and the N123S may be not the key sites N-linked glycosylation modification of LHBs. The N15S and N123S mutations of LHBs contained fucosylated modification sites by Lectin array (Figure 4C). PNGase F converted especially gp42 to the nonglycosylated p39 form of the N320K mutation LHBs (Figure 4D). The N320K may be the key sites N-linked glycosylation modification of LHBs. 

### 4.9. The localization of LHBs and its Mutations in L02 Cells

To demonstrate the localization of LHBs and its mutations in L02 cells, they were cultured then cotransfected with plasmids. The confocal images were acquired using Zeiss 510 META confocal microscope. The images showed that CALR protein (red) was localized in the endoplasmic reticulum (up left, red). LHBs or its mutation (up right, green) was localized on endoplasmic reticulum too. The nuclear of cells (down left, blue) were stained by DAPI. The overlaid images indicated that CALR overlapped with LHBs or its mutation on endoplasmic reticulum. The subcellular localization of LHBs was similar to its mutations (Supplement 2A-2E, A; LHBs; B: N15S; C: N123S; D: N177S; E: N320K).

### 4.10. The Secretion Levels of LHBs and its Mutations

**Figure 5.**
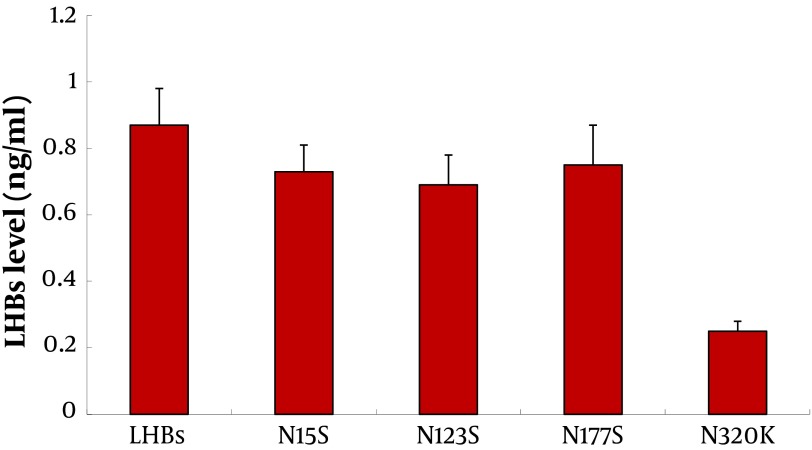
ELISA Analyses for the Secretion of LHBs and Its Mutations L02 cells were transfected with pCDNA3.1 (-), pCDNA3.1 (-)-LHBs, pCDNA3.1(-)-N15S, pCDNA3.1(-)-N123S, pCDNA3.1(-)-N177S, and pCDNA3.1(-)-N320K respectively as described in Materials and Methods. After 48 hrs, the cell culture supernatants were harvested and measured for the release of LHBs by ELISA. **P < 0.01

To demonstrate the secretion of LHBs and its mutations in L02 cells, they were cultured then transfected with plasmids. The LHBs levels in cell culture supernatants were detected by ELISA kit. The results showed that the secretion of LHBs was significantly lower than others in cell culture supernatants (P<0.01) (Figure 5). 

## 5. Discussion

HBV has been considered the crucial cause of HCC. Wang et al. have found that deletions at pre-S1 or pre-S2 of LHBs domains could be observed in ground glass hepatocytes (14). The pre-S mutant LHBs is located at the lumen of ER (22). These mutated LHBs proteins could be detected in serum and HCC tissues of patients (4). It can induce ER stress, and then oxidative DNA damage and genomic instability (23). Moreover, it can promote expression of cyclin A, progression of cell cycle, and multiplication of hepatocytes (15). The pre-S mutants were high risk factors of HCC identified by nested control study (24). Taken together, pre-S mutants of LHBs are the potential occasions in hepatocellular carcinogenesis related to HBV. The association between virus, ER stress and HCC has been confirmed (25).

Our study reported the consensus motifs of LHBs mutants (N15, N123, N177 and N320). Then, we focused on those motifs and occurrence of those putative asparagines residues by serine or lysine. Our results demonstrated that the modification of N-glycosylation of LHBs was related to ER stress, especially in N-glycosylation sites N177S and N320K. The markers of ER stress, GADD34 and GRp78 expression were significantly upregulated by LHBs and its mutations, especially N320K (Figure 1A). It may be the potential role on HBV-related hepatocarcinogenesis associated with ER stress induced by N-glycosylation modification of LHBs. 

We found that the N15S, N123S and N177S mutated LHBs proteins could induce overexpression of EDEM in L02 cells (Figure 1B). The N15S, N123S, N177S and N320K LHBs mutation proteins interacted with EDEM protein in L02 cells, especially in N177S, but not LHBs (Figure 1C). Overexpression of EDEM induced faster release of misfolded proteins and promoted ER-associated degradation (ERAD) ( 20 , 21 ). Misfolded of LHBs could interact with EDEM, and promote its expression. Upregulation of EDEM may promote cell recovery from ER stress originated from LHBs, especially in its mutations. LHBs misfolded proteins in ER could be degraded by overexpression of EDEM. But the interaction between N320K and EDEM may block the ERAD pathway and its secretion (Figure 5). 

Our results confirmed that LHBs and its four mutated proteins contained p62-derived UBA domain, especially in N15S, N123S and N320K mutations. Most studies on p62 have indicated that it could induce inhibition of apoptosis and proteasomal (21). The ubiquitin pathway may be another major non-lysosomal proteolytic pathway of misfolded LHBs proteins, and related to liver cells function of liver cells or HCC.

The research showed that the N15S, N123S and N177S mutated LHBs proteins could induce overexpression of cyclinA in L02 cells. The LHBs and N123S, N177S, N320K LHBs mutation proteins could induce overexpression of cyclin B1 in L02 cells, especially in LHBs. The N15S, N123S and N177S LHBs mutation proteins could induce overexpression of cyclin D1 in L02 cells, especially in N15S and N123S LHBs mutations. The N320K LHBs mutation proteins could induce overexpression of cyclin E (Figure 5). The N-linked glycosylation modification of LHBs affected on expression of cyclins and related to HCC. 

Concanavalin A (ConA) has been used to study characterization of glycoproteins (26). LHBs and its four mutations were the N-glycoproteins by Con A assays. The N15S and N123S mutations of LHBs contained fucosylated modification sites by Lectin array;. PNGase F is an important enzyme, which could be used to split high mannose. N-linked glycoproteins can cleave by it to analyze hybrid and complex oligosaccharides from proteins. PNGase F converted especially gp42 to the nonglycosylated p39 form of the N320K mutation LHBs.

But the subcellular localization of LHBs was similar to its mutations; it indicated that modification of N-glycosylation had no effect on the localization of LHBs. The secretion of LHBs or its mutations were blocked, especially in N320K. LHBs and its mutations induced an increase in G1 phase and inhibition of S phase, especially in N320K (Figure 3A). As shown in Figure 3B, incorporation of BrdU into the control, accumulation of mitotic L02 cells was delayed by LHBs and its mutations, especially in N320K. In conclusion, the N320K may be the key sites N-linked glycosylation modification of LHBs.

## Supporting Information

Supplement 1. The Diagram of Mutated Base and Amino Acid Sequence of LHBs The base (A) and amino acid (B) sequence of N 15 S-LHBs mutated on the point Asn15 of N- glycosylation. The base (C) and amino acid (D) sequence of N 123 S- LHBs mutated on the point Asn123 of N- glycosylation. The base (E) and amino acid (F) sequence of N 177 S -LHBs mutated on the point Asn177 of N- glycosylation.The base (G) and amino acid (H) sequence of N 320 K-LHBs mutated on the point Asn320 of N- glycosylation

Supplement 2. The Localization of LHBs and Its Mutations in L02 Cells To demonstrate the localization of LHBs and its mutations in L02 cells, cells were cultured then co-transfected with plasmids. After 48 hrs, cells were deal with PFA and DAPI, confocal images were acquired using Zeiss 510 META confocal microscope. CALR protein (red) localized to the endoplasmic reticulum (upper left, red). LHBs or its mutation (upper right, green) localized on endoplasmic reticulum too. The nuclear of cells (down left, blue) were stained by DAPI. The overlaied images indicated that CALR overlapped with LHBs or its mutation in endoplasmic reticulum (down right). LHBs (A), N15S (B), N123S (C), N177S (D) and N320K (E)
